# NIRS-based monitoring of kidney graft perfusion

**DOI:** 10.1371/journal.pone.0243154

**Published:** 2020-12-02

**Authors:** Stepan Maly, Libor Janousek, Radoslav Bortel, Jan Sebek, Jiri Hospodka, Jiri Skapa, Jiri Fronek

**Affiliations:** 1 Transplant Surgery Department, Institute for Clinical and Experimental Medicine, Prague, Czech Republic; 2 First Faculty of Medicine, Charles University in Prague, Prague, Czech Republic; 3 Faculty of Electrical Engineering, Czech Technical University in Prague, Prague, Czech Republic; 4 Department of Anatomy, Second Faculty of Medicine, Charles University in Prague, Prague, Czech Republic; Politecnico di Milano, ITALY

## Abstract

**Introduction:**

Acute early vascular complications are rare, but serious complications after kidney transplantation. They often result in graft loss. For this reason, shortening the diagnostic process is crucial. Currently, it is standard procedure to monitor renal graft perfusion using Doppler ultrasound (DU). With respect to acute vascular complications, the main disadvantage of this type of examination is its periodicity. It would be of great benefit if graft blood perfusion could be monitored continuously during the early postoperative period. It appears evident that a well-designed near infrared spectroscopy (NIRS) monitoring system could prove very useful during the early post-transplantation period. Its role in the immediate diagnosis of vascular complications could result in a significant increase in graft salvage, thus improving the patient’s overall quality of life and lowering morbidity and mortality for renal graft recipients. The aim of this study was to design, construct and test such a monitoring system.

**Materials and methods:**

We designed a rough NIRS-based system prototype and prepared a two-stage laboratory experiment based on a laboratory pig model. In the first stage, a total of 10 animals were used to verify and optimize the technical aspects and functionality of the prototype sensor by testing it on the animal kidneys in-vivo. As a result of these tests, a more specific prototype was designed. During the second stage, we prepared a unique laboratory model of a pig kidney autotransplantation and tested the system for long-term functionality on a group of 20 animals. Overall sensitivity and specificity were calculated, and a final prototype was prepared and completed with its own analytic software and chassis.

**Results:**

We designed and constructed a NIRS-based system for kidney graft perfusion monitoring. The measurement system provided reliable performance and 100% sensitivity when detecting acute diminished blood perfusion of the transplanted kidneys in laboratory conditions.

**Conclusion:**

The system appears to be a useful tool for diagnosing diminished blood perfusion of kidney transplants during the early postoperative period. However, further testing is still required. We believe that applying our method in current human transplantation medicine is feasible, and we are confident that our prototype is ready for human testing.

## Introduction

Kidney transplantation is a standard medical procedure for patients suffering from chronic renal failure. In comparison with hemodialysis or peritoneal dialysis, it provides patients with better quality of life and a longer overall survival rate.

Due to the shortage of suitable grafts worldwide, excellent long-term performance of the grafts is essential. During the early post-transplant period, sufficient and uninterrupted perfusion by arterial blood, as well as unhindered outflow of venous blood, is necessary for long-term graft survival and performance. Complications in this regard can cause delayed graft function, chronic graft dysfunction and, in extreme cases, primary a function and graft loss.

Early vascular complications are among rare complications that can arise during the post-transplant period [1, 2]. Vascular complications can have severe consequences [1, 2], which may include arterial and venous thrombosis, renal artery stenosis, renal artery kinking and renal artery laceration or dissection. Acute surgical re-intervention is almost always indicated in these cases. Even short delays in administering treatment may cause extensive damage to the graft and expose the patient to the risk of graft failure. For this reason, it is crucial to shorten the diagnostic process to a minimum.

Currently, it is standard procedure to monitor renal graft perfusion using non-invasive Doppler ultrasound [3–5], which is a widely available method. It does, however, have its limitations. With respect to acute vascular complications, the main disadvantage of this examination is its periodicity.

It would be of great benefit if graft blood perfusion could be monitored continuously during the early postoperative period. In literature, two approaches are recommended when continuously monitoring kidney graft perfusion: measuring of electromagnetic waves with near-infrared spectroscopy (NIRS) [6–8] and monitoring mechanical waves through Doppler ultrasound (DUS) [9, 10].

Doppler flow probe (DFP) uses ultrasound waves to monitor vascular flow and blood perfusion. However, this method has many disadvantages.

In transplantation medicine NIRS has been used in experimental pediatric post-transplant monitoring, but only as a non-invasive method [6–8]. This limits the usage to only children and adults with slim builds. Nevertheless, the method can also be used invasively [11, 12]. This way, the method can be used for all transplant recipients and give us reliable information.

To our knowledge, there are currently no available invasive NIRS based measurement systems that would allow continuous monitoring of kidney graft perfusion during the postoperative period. It appears that a well-designed NIRS monitoring system could prove very useful in the early post-transplantation period. Its role in the immediate diagnosis of vascular complications could result in a significant increase in graft salvage, thus improving the patient’s overall quality of life and lowering morbidity and mortality rates.

The aim of this experiment was to design, construct and test such a monitoring system.

## Materials and methods

### Ethical statement

This study was reviewed and approved by the Ministry of Health of the Czech Republic—num. 29/2016.

All legal and ethical requirements for this animal laboratory experiment have been met.

The Institute for Clinical and Experimental Medicine, Prague, Czech Republic (IKEM), is in possession of an active license, which enables it to take part in experimental work on laboratory animals.

All transplantations and procedures performed on laboratory animals were carried out in an operating theatre under the care of a trained anesthesiologist. After surgery, all animals were administered sufficient analgesic drugs. All animals were euthanized under full anesthesia at the end of the experiment.

## Experiment

Based on previous studies [6–8, 11, 12] and our own unpublished experiments, we assumed that kidney graft blood perfusion can be continuously monitored using visible and near-infrared light spectroscopy.

We prepared a rough NIRS measuring system prototype. The system was powered by its own energy source and was able to transmit wireless data directly to a secondary monitoring computer. The system was able to emit and monitor a broad spectrum of visible and near-infrared electromagnetic waves.

We prepared a laboratory project based on a laboratory pig models. In-vivo testing was divided into two stages and three subgroups of laboratory animals.

We chose laboratory pigs for our study for several reasons. The pig kidney is very similar to that of a human in anatomy and physiology [13, 14]. These similarities extend to vascular supply, capsule structure and organ shape [15, 16]. We selected animals weighing approximately 40kg since their organs are similar in size to those of humans. Their internal anatomy and organ placement are also similar to those of humans, allowing us to mimic the physical conditions of human kidney transplantation.

The surgery took place in a specialized surgical theatre. After sedation using Intramuscular injection of *Ketamine+Azaperone+Atropine*, full anesthesia was induced by i.v. bolus of *Propofol+Fentanyl*. The animal was placed on the operating table with machine ventilation. Continuous anesthesia was maintained by inhalation of Isoflurane and continuous intravenous infusion of Fentanyl. For relaxation intravenous bolus of *Pipecuronium Bromide* was applied. The animal was given *Amoxycilline*(875mg/125mg) with an enzyme inhibitor at the beginning of every procedure. During the surgical procedure we continuously monitored; arterial pressure, ECG, and saturation (tongue/lip). In the final step, the animal was euthanized in full anesthesia using bolus *Thiopental* (1-2g) + *KCl* 7,45% solution (40–50ml) i.v.

All drug dosages were calculated based on actual animal weight.

Stage I
  The first group (6 animals) was utilized to verify and optimize the technical aspects and functionality of the prototype sensor. In Stage I, we used hardware measuring at 7 wavelengths (465, 560, 650, 740, 850, 940 and 1050nm).

The animal kidney was approached extraperitoneally using midline laparotomy, and the vessel supply of kidney was prepared for clamping. The prototype sensor was placed on the ventral plane of the kidney parenchyma. The sensor almost reached from one pole of the kidney to the other. This way, we could monitor the graft in its entire length.

**Fig 1:**
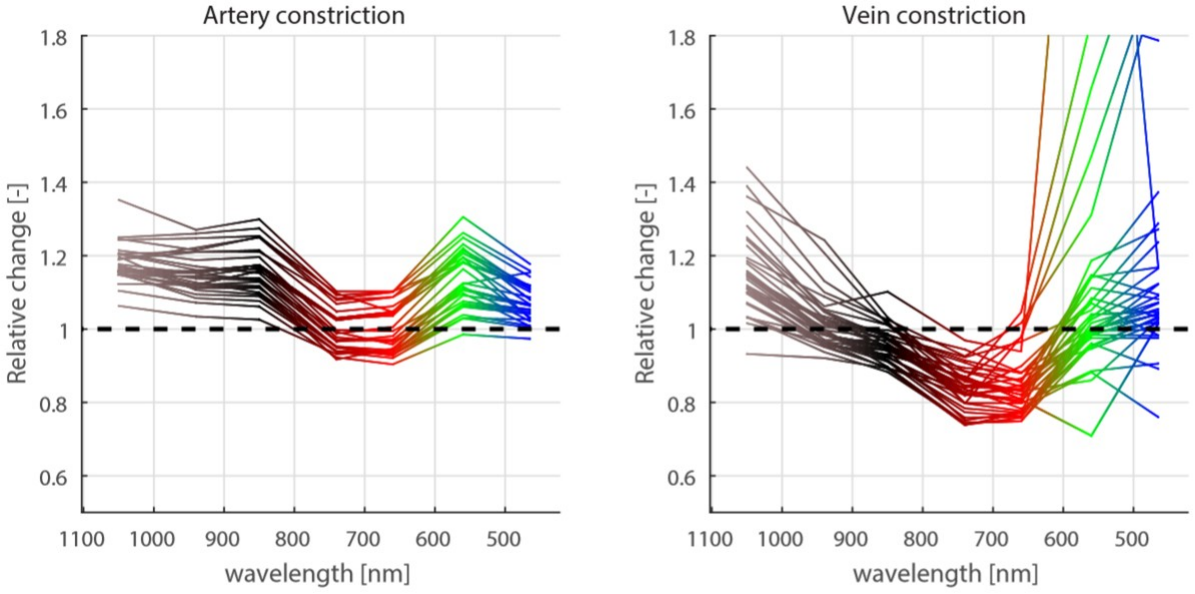
Monitored wavelength changes from baseline in case of artery and vein iatrogenic obstruction.

The kidney was then put through a series of cycles, during which kidney blood flow (arterial or venous) was obstructed (fully or partially) for 10 minutes and later returned to normal. During testing, the quantities were recorded and evaluated. Fig 1 indicates the relative changes that were measured at individual wavelengths.

The second group (4 animals) was utilized to establish and optimize sensor placement in the closed wound and to verify its function reliability over an extended period of time (48 hours).

The animal kidney was approached extraperitoneally using midline laparotomy. The prototype sensor was placed on the exposed kidney capsule, located on the ventral plane of the organ. The system was fixed to the animal and the wound was closed. The animal was then allowed to move freely for 48 hours. During testing, the quantities were recorded and evaluated.

**Fig 2:**
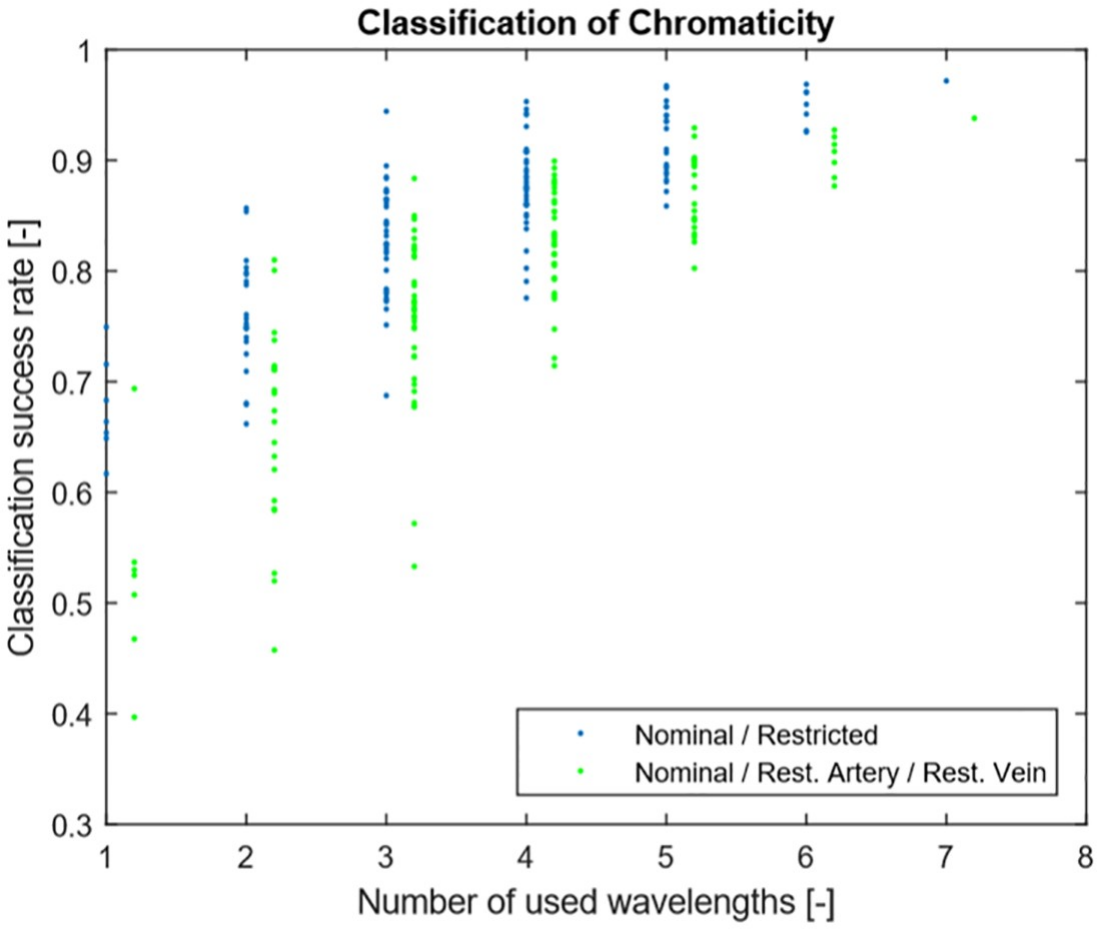
Classification of Chromaticity. Classification of Chromaticity—Detected Classification success Nominal/Restricted (blue) and Classification success Nominal/Restricted Artery/Restricted Vein (green) based on number of wavelengths included into the calculation.

Data taken from the first and the second group were used to optimize the measurement hardware setup, primarily to select a suitable subset of wavelengths. To simulate data that could be measured during artery/vein occlusion on a freely-moving animal, we added signals taken from both the first group (i.e. data from Fig 1) and the second group. Consequently, the resulting data included the effects of both blood vessel obstruction and motion artifacts. These combined signals were then used to train and test a classifier (primarily the quadratic classifier; the k-nearest neighbor and the Parzen window classifiers were also tested, providing essentially the same results). The classification success rate was evaluated for all distinct combinations of wavelengths (Fig 2). Based on these results, we chose the best performing set of four wavelengths (650nm, 740nm, 850nm and 940nm). Four light emitting diodes can be integrated to a reasonable space (they require a semi rigid printed circuit board 6.7mm wide, and the connection to the measurement electronics can be provided by a similarly sized 6mm wide flat flexible cable), while they provide a reasonable classification success rate (this was further confirmed by the evaluation in Stage II).

Stage II
  The final group (20 animals) was utilized to simulate transplantation perioperative and early postoperative conditions and to verify system functionality and efficiency.

We utilized surgical experience, gained from the first stage of the experiment, in the preparation of a unique laboratory porcine model of kidney transplantation. We performed an ipsilateral kidney autotransplantation to the iliac fossa using the retroperitoneal approach—Mimicking the conditions of human transplantation. The transplantation was completed with a standard full-thickness ureteroneocystostomy. The prototype sensor strap was then applied to the ventral plane of the graft. The connecting cord was passed through the abdominal wall (as would be done with drainage), and the battery and transmitter were fixed to the back of the animal. We fixed a second sensory system to the contralateral kidney in order to compare the performance of the transplanted graft and native kidney, thus eliminating any system perfusion complications.

We then monitored the perfusion and effects of the wound oppression during closure (even simulating over-oppression).

Two types of measurements were performed on each of the 20 laboratory animals.

The first type was a 48 hour measurement after transplantation. During this period, the animal was in an awakened state and moving freely.

The second type was a short-term (10+10 minute) measurement. The animal was put under anaesthesia and underwent arterial or venous clamping of the graft, which lasted 10 minutes. Subsequent changes in the measured wavelengths were recorded and analyzed and the blood flow was restored for another 10 minutes. These cycles were repeated to obtain more data.

Prior to the animal being euthanized, we removed the sensor in order to verify that such action would not cause any complications, such as bleeding and tissue damage.

All the data were analyzed, and a final prototype was prepared and completed with its own analytics software and chassis.

### Technical description

**Fig 3:**
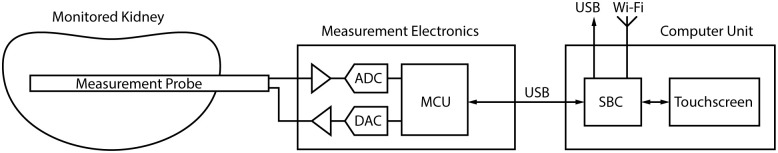
Measurement system technical schemata. ***SBC—***Single Board Computer, ***MCU***—Microcontroller Unit, ***ADC***—Analog to Digital Converter, ***DAC***—Digital to Analog converter.

**Fig 4:**
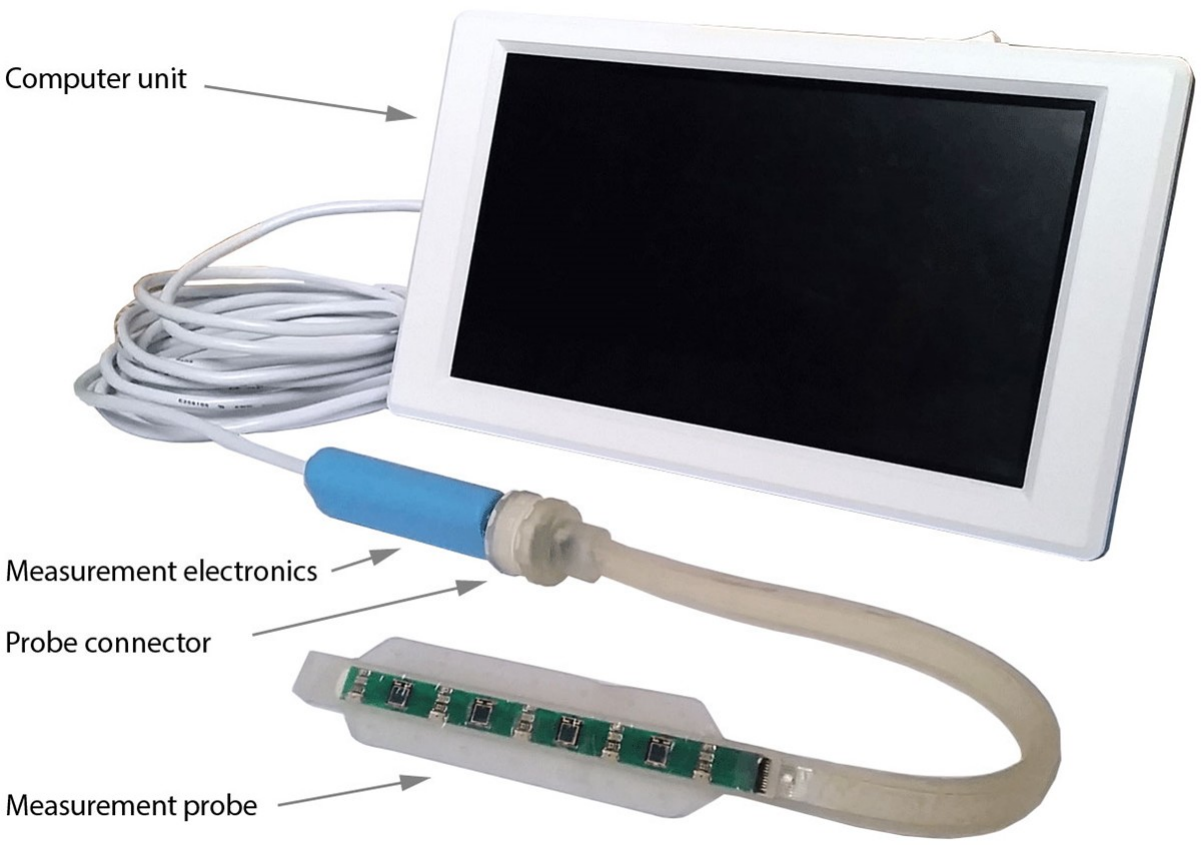
Measurement system.

The final kidney graft monitoring system (Figs 3 and 4) comprises a measurement probe (placed directly over the length of the ventral plane of the kidney), measurement electronics, and a computer unit.

The measurement probe contains four measuring points, each equipped with light sources of four wavelengths (650nm, 740nm, 850nm and 940nm) and a photodiode that captures transmitted/reflected light. Specifically, the light emitters are HSMH-C170, MTSM0074-843-IR, VSMY1850 and APT1608F3C (the plastic lens of MTSM0074-843-IR was mechanically removed in order to decrease the height of the sensor). The photodiode is TEMD5010X01. The measurement at different wavelengths is performed using a time division multiplex with sampling frequency at each wavelength equal to 25Hz. During each sampling period, the individual LEDs are sequentially activated, and the intensity of reflected/transmitted light is measured at each wavelength separately. The RMS/maximal currents through the individual diodes were 308/1500, 154/750, 308/1500, 1230/6000 [*μA*], at 650nm, 740nm, 850nm and 940nm, respectively. Every measuring point thus provides four signals, each corresponding to a single wavelength. The group of signals provided by a single measurement point is referred to as a channel. The arrangement with four measuring points allows better coverage of kidney graft volume and allows exclusion of unsuitable channels.

The measurement electronics generates signals for the light sources and captures weak signals from the photodiodes. It amplifies and digitizes them, and transfers the data to the central computer unit via a USB connection. The measurement probe sensor and the measurement electronics are encapsulated in epoxy and silicone (hardness Shore A 33).

**Fig 5:**
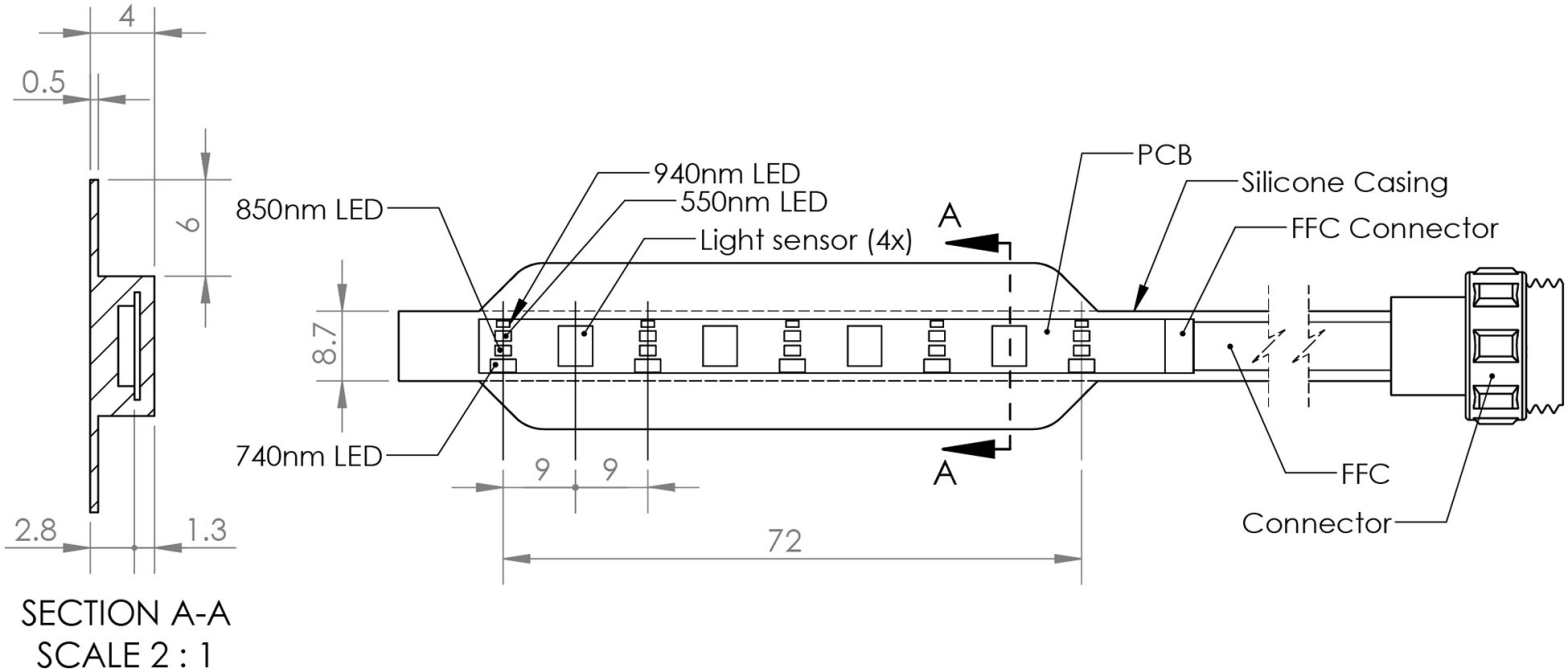
Drawing of the measurement probe. ***FFC***—Flat Flexible Cable, ***PCB***—Printed Circuit Board, ***LED***—Light Emitting Diode.

**Fig 6:**
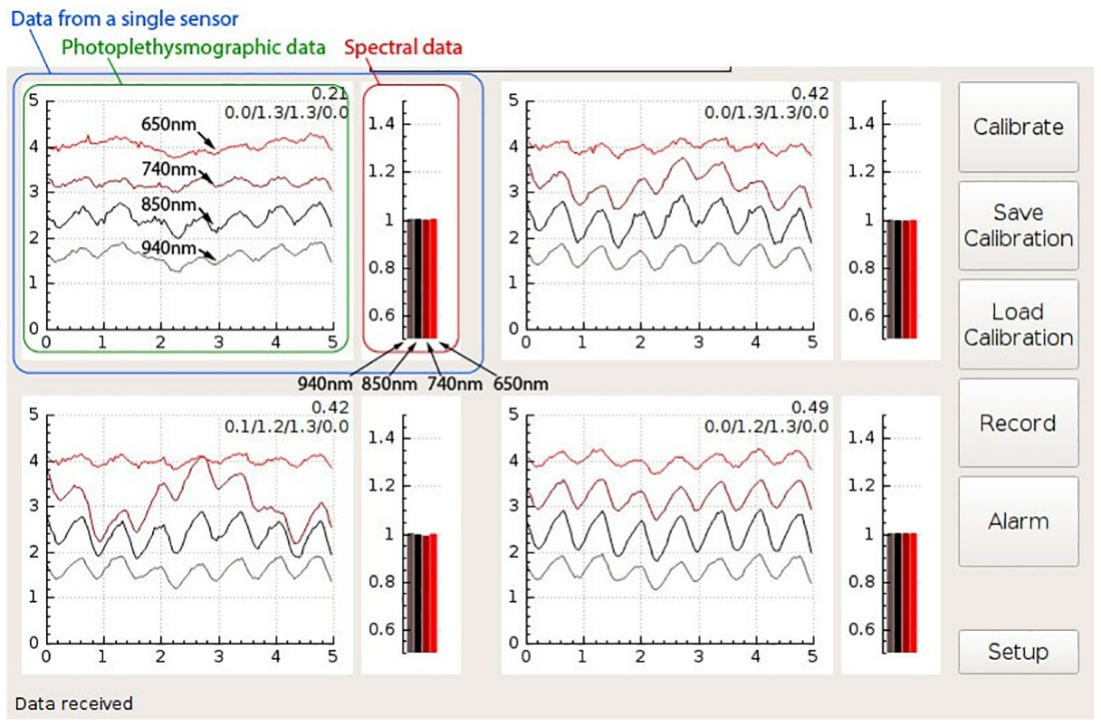
A screenshot of the GUI during the monitoring of a kidney with unrestricted blood flow. The GUI shows signals from four channels, each monitoring at four wavelengths. The meaning of the individual plots is described for the channel shown on the top left side. The baseline wonder of the 740nm intensity visible in the bottom left channel is most likely a motion artifact caused by breathing (The 740nm wavelength is the most effected, as the corresponding LED is positioned at the outer edge of the measurement probe, which is usually the most the prone to the motion artifacts). The numbers at the top right of each channel plot are internal algorithm values kept on the prototype system for debugging purposes, and would not be present on a production run GUI (the reader can ignore these values).

**Fig 7:**
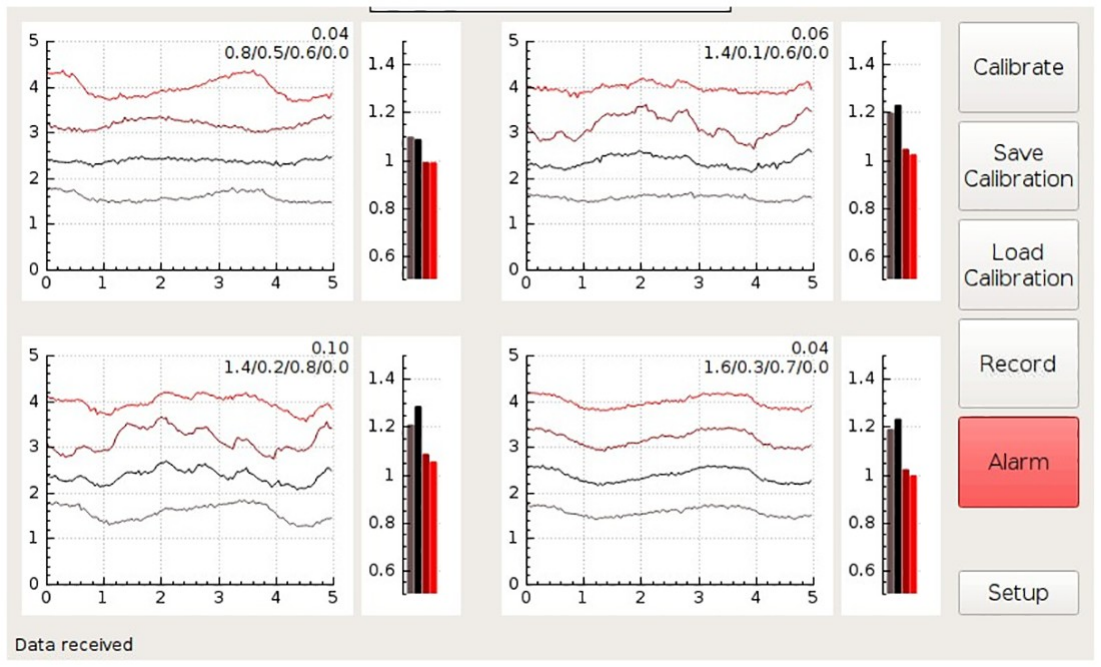
A screenshot of the GUI during the monitoring of a kidney with constricted renal artery. The baseline wonder observed in some channels is most likely a motion artifact caused by breathing.

**Fig 8:**
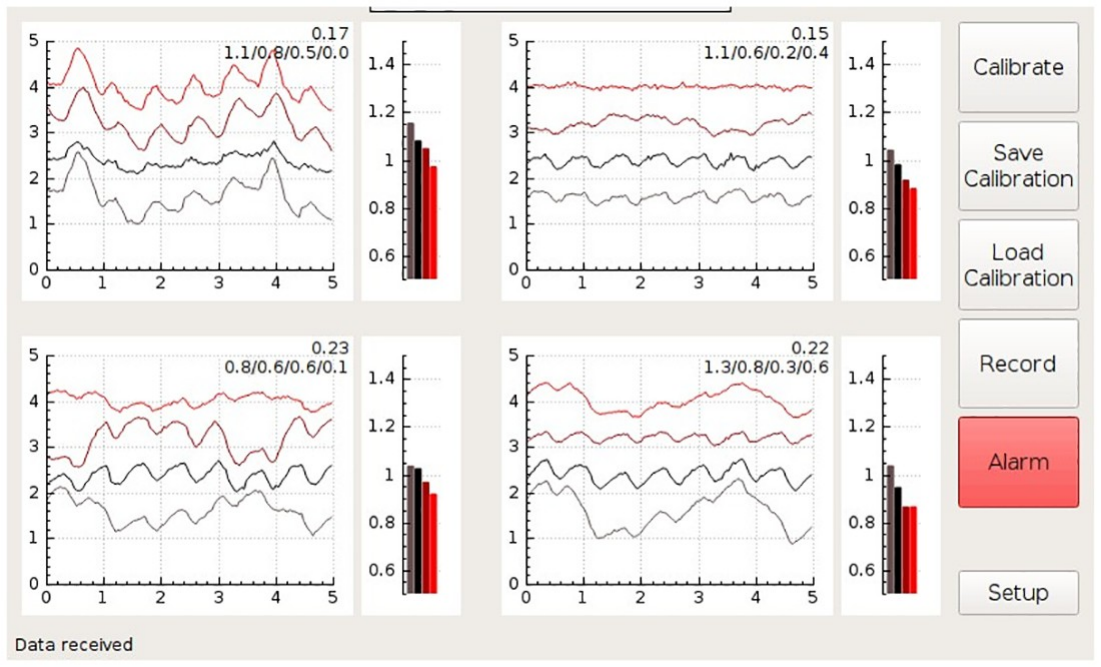
A screenshot of the GUI during the monitoring of a kidney with constricted renal vein. Immediately after vein constriction the pulsatile waveforms are typically present; however, these do not necessarily reflect only changes in transmittance/reflectivity. They can also be affected by the motion artifacts created by the arterial pulse wave. The baseline wonder observed in some channels is most likely motion artifact caused by breathing.

The computer unit houses a single board computer with a touch screen. It provides USB and Wi-Fi connectivity for remote control and data access. The single-board computer runs a Linux operating system and custom-developed software that acquires data from the measurement electronics and classifies the state of the monitored kidney. The results are presented on the touch screen via a graphical user interface that visualizes the spectral properties and photoplethysmographic waveforms of the organ (Figs 5–8).

### Data analysis

#### Signal processing

The measured signals were divided into two groups: slow (<0.1Hz) changes that reflect the overall spectral properties of the transplanted kidney and faster (>0.3Hz) changes that show pulsatile signals related to the variations of blood oxygenation during a heart cycle. The low pass and high pass filters were implemented as 3^rd^ order Chebyshev filters.

The fast, high frequency signals with pulsatile changes can be interpreted as photoplethysmographic signals, and they are known to provide simple means of monitoring blood oxygen saturation. However, it was evident to us that these signals are highly susceptible to motion artifacts (including body movement, breathing and even hearth action). These artifacts affect the optical coupling between the probe and the monitored tissue, and can affect the intensities of the measured transmitted/reflected light. This problem is more pronounced in our application where the probe is not firmly attached to the monitored tissue (in contrast, a pulse oximeter attached by a clip would achieve a much more stable optical coupling; therefore, motion artifacts would be much less of an issue). The motion artifacts can either render the pulsatile waveform undistinguishable (typically during dynamic movement of the animal), they can cause mild baseline wonder (typically due to breathing), or they can affect the amplitudes of the pulsatile waveforms (e.g. due to an arterial pulse wave). Consequently, we determined that these signals are not sufficiently reliable to provide robust monitoring of blood perfusion. Even though these signals are monitored and visualized by our system, their primary use is limited to the initial setup of the measurement. Shortly after the kidney graft transplant (when the animal was strictly limited in movement) the presence of the pulsatile waveform can indicate which channels can be used for further monitoring; if no pulsatile changes are observed in a given channel, it is excluded from further monitoring.

The slower, low frequency signals reflect the optical properties of the kidney graft on a time scale greater than the duration of a heartbeat. We found this to be a much more reliable indicator of kidney graft blood perfusion. We observed that the changes in these signals, caused by the decrease in the blood perfusion, is about one order of magnitude greater than the changes observable in higher frequency pulsatile waveforms. Consequently, the monitoring of slow changes is much less susceptible to the motion artifacts. We found that mechanical movement can still cause simultaneous increases and decreases in intensities in all channels; however, this effect is small enough to be effectively suppressed by normalizing the measurements with respect to the intensity measured at 940nm. Consequently, we based further classification on the slow changes in intensities measured at 650nm, 740nm and 850nm, normalized relatively to the intensity at 940nm (any other wavelength can be used for normalization without diminishing the results). The normalization was performed by dividing the measured intensities at 650nm, 740nm and 850nm by the intensity at 940nm.

Before the data were fed to the classifier, we computed their natural logarithm. Thus, the classified feature vector consisted of three numbers that were natural logarithms of normalized intensity at three wavelengths.

**Fig 9:**
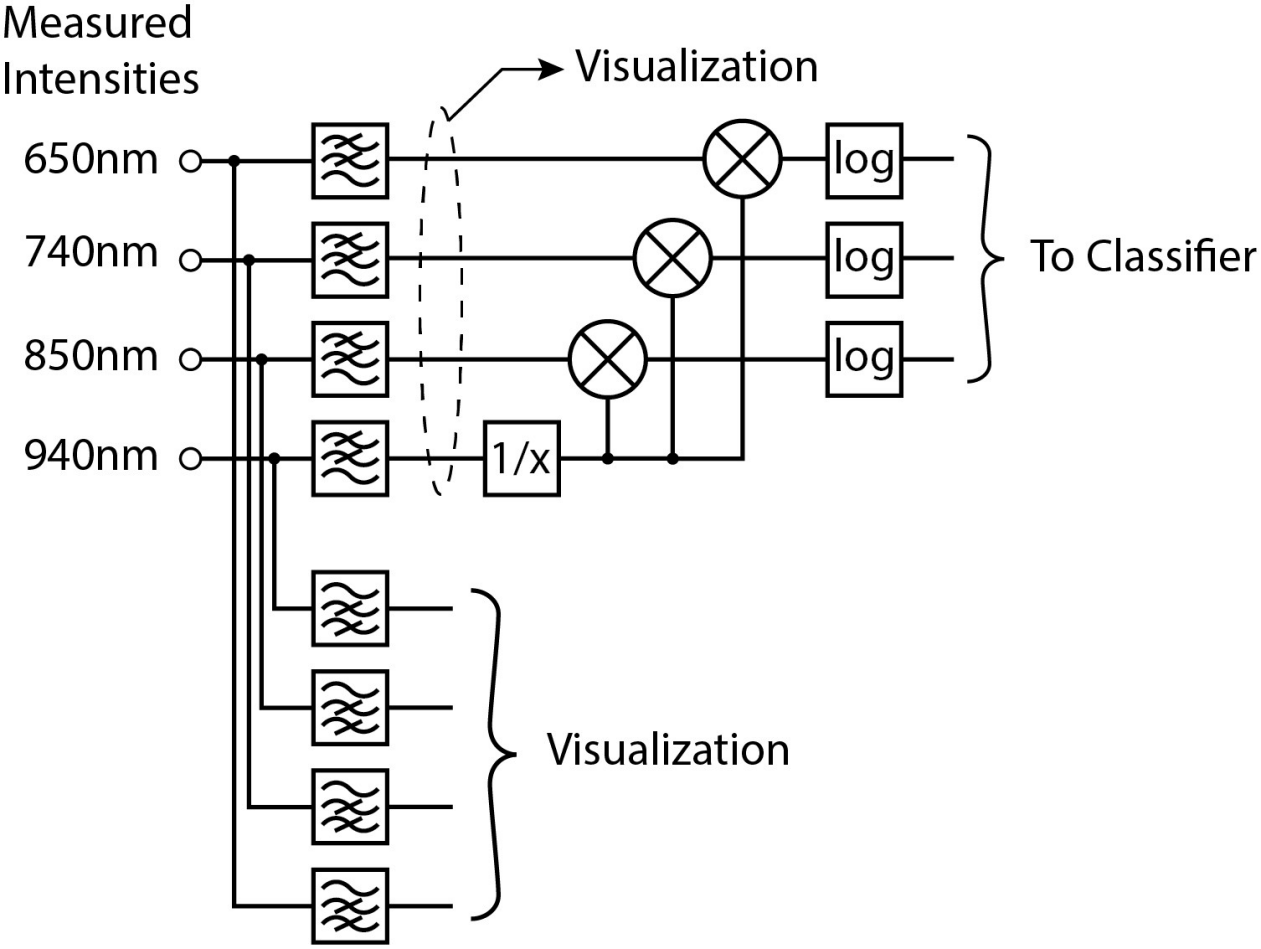
Block diagram of signal pre-processing.

The block diagram of the signal pre-processing path is shown in Fig 9.

#### Classification

After initial processing, the data were subjected to the classification of detecting diminished blood perfusion. The following subsections describe the main elements of the classifier.

*Training data*. To train the classifier, we used data from both the long-term and short-term measurements. These data were sufficient, so to avoid unnecessary overtraining, the long term measurements, during which the transplanted kidney failed, were not used in training.

*Testing data*. All of the above-mentioned data were used in testing. To avoid overlearning, the leave-one-out cross-validation was used: to evaluate the performance on a chosen measurement, data obtained during this measurement were excluded from a training set. The classifier was retrained using the remaining data, and only then was it applied to the previously excluded measurement, evaluating its success rate.

*Classifier construction*. To classify blood perfusion, we used a Bayesian classifier [17]. The probability density functions (PDF) of the classified data were approximated using a Gaussian mixture model (GMM), with 20 mixture components, using the data from long-term measurements with correctly functioning kidneys. In cases where data were obtained from short-term measurements (i.e. measurements where veins and arteries were mechanically occluded), we approximated PDFs using the kernel density estimation (KDE) with a Gaussian kernel. We selected the kernel density estimation because of its ability to approximate more complex probability density functions. However, in the case of long-term measurements, there is a large amount of data, which makes a KDE based classifier computationally intensive. We therefore chose a GMM in its place.

The classifier was constructed to identify 3 classes:

1. correct blood perfusion
2. diminished blood perfusion due to a vein obstruction
3. decreased blood perfusion due to an artery obstruction

To avoid false-positive detections of diminished blood perfusion due to sensor movement or other short-term interferences, the classifier initiates a warning only if diminished blood perfusion is classified continuously for more than 3 minutes.

## Results

**Fig 10:**
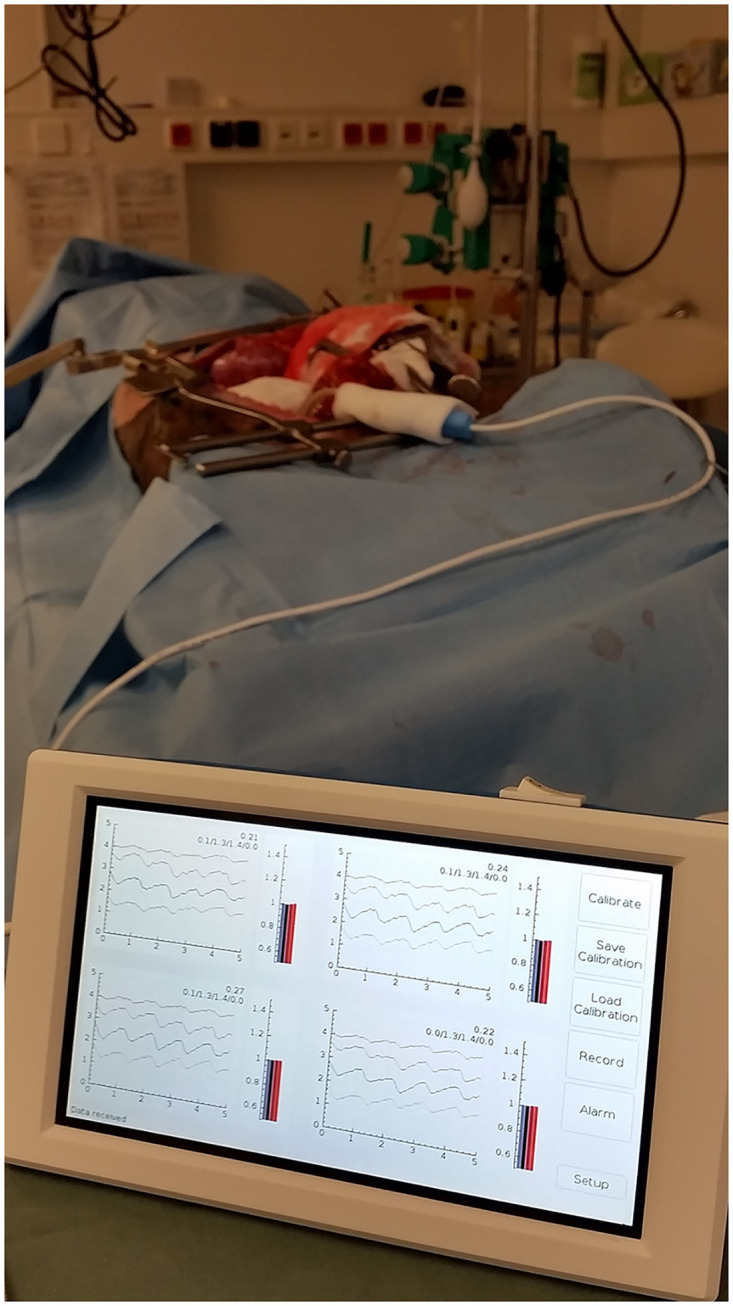
Measurement system in use on a porcine kidney transplant.

We designed and constructed a new NIRS-based measurement system for kidney graft perfusion monitoring (Fig 10).

We managed to obtain a total of 14 long-term measurement data sets (Table 1). In other cases, data were lost due to a technical failure or an acute thrombosis of the graft, which occurred during monitoring (all of these failures were correctly recognized by the system).

**Table 1:** Data analysis—14 long-term (48hours) renal graft perfusion measurements.

Vein obstruction FPR	0.20%
Artery obstruction FPR	0.30%
Combined FPR	0.30%

***Vein obstruction false positive rate (FPR)***—Rate of false detection of diminished blood perfusion due to vein obstruction, ***Artery obstruction false positive rate (FPR)***—Rate of false detection of diminished blood perfusion due to artery obstruction, ***Combined false positive rate (FPR)***—Rate of false detection of diminished blood perfusion due to either artery or vein obstruction

For the short-term data, we obtained 18 artery obstruction and 16 vein obstruction data sets (Table 2).

**Table 2:** Data analysis—Short-term measurements—Latrogenic clamping of renal graft arteries or veins.

	n	Detection FNR	Classification FNR	sensitivity
Artery obstruction	18	0.0%	5,6%	100.0%
Vein obstruction	16	0.0%	31,3%	100.0%

***n***-number of clamping cycles, ***Detection false negative rate (FNR)***—Percentage of cases where the classifier did not correctly indicate diminished blood perfusion (arterial x venous), ***Classification false negative rate (FNR)—***Percentage of cases in which the classifier failed to indicate correct cause of diminished blood perfusion (i.e. classifier incorrectly indicated artery obstruction in case of vein obstruction and vice versa), ***Sensitivity—***Percentage of cases where the classifier did correctly indicate diminished blood perfusion

The measurement system manifested reliable performance in detecting diminished blood perfusion of transplanted kidneys.

For long-term measurements, where the transplanted kidney performed correctly, the classifier showed a low false-positive rate (Table 1). In cases where the alarm was initiated only when blood perfusion was diminished for more than 3 minutes, the number of false alarms was even lower. Furthermore, if the alarm would be accepted if it was detected in at least two channels simultaneously, our long-term measurements would be without any false indications of low blood perfusion.

For long-term measurements, where transplanted kidney perfusion became obstructed, the classifier correctly identified all failures.

For short-term measurements, intensity changes induced by vessel clamping were classified correctly (Table 2). There was a relatively high rate of error when identifying the cause of diminished blood perfusion (i.e. occlusion of renal artery vs renal vein); however, this distinction is not vital for early diagnosis.

The system proved to be safe for the laboratory animal. We did not encounter any complications with our experimental animals.

The significance of the resulting classifier metrics can be verified by statistical testing. The individual metrics can be treated as statistics, and their respective p-values can be computed. We performed this computation using permutation tests [18, 19]. The individual conditions (unrestricted blood flow, artery obstruction and vein obstruction) were considered to be various treatments, and the null hypotheses were that the distributions of the statistics are invariant to the treatments. To perform the permutation tests we repeatedly permuted the feature vectors between the individual treatment groups, and for each permutation we retrained and retested the classifier. 1000 permutation was used. The FPR, FNR obtained for each permutation were collected into empirical distributions, the quantiles of which were then used to estimate the individual p-values. For the vein obstruction FPR, artery obstruction FPR, combined FPR, artery obstruction detection FNR, vein obstruction detection FNR and artery obstruction classification FNR we obtained p = 0.001 (this is a conservative estimate; for a higher number of permutation a lower p-value could be obtained). For vein obstruction classification FNR we obtained p = 0.102. These results show that the classifier’s ability to distinguish between the restricted and unrestricted blood flow is very unlikely to be a mere random occurrence (note that the presented p-values are uncorrected, but even if a simple Bonferroni correction was applied the evidence would still be strong).

## Discussion

Having a means for an acute diagnosis of early vascular complications in the post-transplant period could improve overall results of kidney transplantation.

Two approaches are recommended for the continuous monitoring of kidney graft perfusion: measuring of mechanical waves through Doppler ultrasound (DUS) and monitoring electromagnetic waves with near-infrared spectroscopy (NIRS).

Blood flow can be continually measured using DUS with an invasive Doppler flow probe (DFP). This is currently an accepted method that is growing more common every year, even in transplantation medicine [9, 10]. This method, however, has many disadvantages [20]. The probe requires a vessel attachment, which may result in iatrogenic vascular complications caused by the sensor itself. The risk of such an incident is low owing to the utilization of modern all-soft probes. The soft probe head still needs to be wound around the artery and even leave foreign synthetic material on the vessel after the probe head has been removed. Moreover, a single probe can monitor only a single vessel at a time, giving us only partial information of the overall graft perfusion [20].

Experimentally, NIRS has been used to monitor organ perfusion (even non-parenchymatous) [21] and even considered in post-transplant graft monitoring [22]. In human transplantation medicine, NIRS has been used in pediatric post-transplant monitoring, but only as a non-invasive method [6–8]. That can only be considered in children and adults with slim builds. Nevertheless, the method can also be used invasively [11, 12]. This way, the method can be used for all transplant recipients and give us reliable information. As with a DFP, this also requires close contact between the probe head and the graft. However, in this case, the sensor is placed in a much safer place—the kidney parenchyma. Only one sensor is needed to both detect complications in arterial and venous vessels, and differentiate between the two.

When compared to a DFP, NIRS monitoring could prove to be a safer, cheaper and more specific solution.

We believe our system would be of benefit, especially in situations with high rates of vascular complications (e.g., complex graft anatomy, re-transplantation, or prothrombotic disorders).

In the current stage presented study limitations are: an animal laboratory study results may vary a little, when implemented into human medicine. This being our initial study, we decided to use smaller group of animals to verify our systems functionality. When aiming for more significant statistical results, we will need to expand the cohort. Technically, our materials will need to be approved for human testing and long term in-vivo safety.

## Conclusion

Under laboratory conditions, our NIRS monitoring system proved reliable, with a very high degree of sensitivity as well as favorable specificity between arterial and venous causes.

The system can be a useful tool for providing an early diagnosis of diminished blood perfusion of kidney transplants during the early postoperative period. However, further testing is still needed. We believe the application of our method in current human transplantation medicine is feasible, and our prototype is prepared for human testing.

A similar system might also be of great use in various fields of surgery (e.g., transplantation of other parenchymatous organs, muscle flap transfers, etc.).

## Supporting information

S1 File Arrive. (DOCX)
