# Supplementary material for: NIRS-based monitoring of kidney graft perfusion
Source: PLoS One. 2020 Dec 2;15(12):e0243154. doi: 10.1371/journal.pone.0243154 (PMC7710057; doi:10.1371/journal.pone.0243154)
Supplement: S1 File — (DOCX) [file pone.0243154.s001.docx]

**ARRIVE**

**TITLE**

NIRS-based monitoring of kidney graft perfusion

**ABSTRACT**

**Introduction:** Acute early vascular complications are rare, but serious complications after kidney transplantation. They often result in graft loss. For this reason, shortening the diagnostic process is crucial. Currently, it is standard procedure to monitor renal graft perfusion using Doppler ultrasound (DU). With respect to acute vascular complications, the main disadvantage of this type of examination is its periodicity. It would be of great benefit if graft blood perfusion could be monitored continuously during the early postoperative period.

It appears evident that a well-designed near infrared spectroscopy (NIRS) monitoring system could prove very useful during the early post-transplantation period. Its role in the immediate diagnosis of vascular complications could result in a significant increase in graft salvage, thus improving the patient's overall quality of life and lowering morbidity and mortality for renal graft recipients.

The aim of this study was to design, construct and test such a monitoring system.

**Materials and Methods:** We designed a rough NIRS-based system prototype and prepared a two-stage laboratory experiment based on a laboratory pig model.

In the first stage, a total of 10 animals were used to verify and optimize the technical aspects and functionality of the prototype sensor by testing it on the animal kidneys in-vivo. As a result of these tests, a more specific prototype was designed.

During the second stage, we prepared a unique laboratory model of a pig kidney autotransplantation and tested the system for long-term functionality on a group of 20 animals.

Overall sensitivity and specificity were calculated, and a final prototype was prepared and completed with its own analytic software and chassis.

**Results:** We designed and constructed a NIRS-based system for kidney graft perfusion monitoring. The measurement system provided reliable performance and 100% sensitivity when detecting acute diminished blood perfusion of the transplanted kidneys in laboratory conditions.

**Conclusion:** The system appears to be a useful tool for diagnosing diminished blood perfusion of kidney transplants during the early postoperative period. However, further testing is still required. We believe that applying our method in current human transplantation medicine is feasible, and we are confident that our prototype is ready for human testing.

**BACKGROUND**

Two approaches are recommended for the continuous monitoring of kidney graft perfusion: Doppler ultrasound (DUS) and near-infrared spectroscopy (NIRS).

DUS is already an accepted method that is growing more common every year, even in transplantation medicine [1,2]. This method, however, has many disadvantages [3].

NIRS has been used in experimental pediatric post-transplant monitoring, but only as a non-invasive method [4,5,6]. This approach has severe limitations. Nevertheless, the method can also be used invasively [7,8] to minimize the disadvantages.

When compared to a DFP, NIRS monitoring could prove to be a safer, cheaper and more specific solution.

1. Crane J, Hakim N. The use of an implantable Doppler flow probe in kidney transplantation: first report in the literature. Exp Clin Transplant. 2011 Apr;9(2):118-20.

2. Hakim DN, Nader MA, Sood A, Kandilis A, Hakim NS. Rescue of Transplanted Kidney Thanks to an Implantable Doppler Probe: Is This the Future? Exp Clin Transplant. 2016 Aug;14(4):454-5.

3. Amdisen C, Jespersen B, Møldrup U, Keller AK. The unsuitability of implantable Doppler probes for the early detection of renal vascular complications - a porcine model for prevention of renal transplant loss. PLoS One. 2017 May 25;12(5):e0178301

4. Malakasioti G, Marks SD, Watson T, Williams F, Taylor-Allkins M, Mamode N, Morgan J, et al. Continuous monitoring of kidney transplant perfusion with near-infrared spectroscopy. Nephrol Dial Transplant. 2018 Oct 1;33(10):1863-1869.

5. Vidal E, Amigoni A, Brugnolaro V, Ghirardo G, Gamba P, Pettenazzo A, et al. Near-infrared spectroscopy as continuous real-time monitoring for kidney graft perfusion. Pediatr Nephrol. 2014 May;29(5):909-14.

6. Perez Civantos DV, Munoz Cantero A, Robles Marcos M, Farinas Seijas H, Santiago Trivino MA, Perez Frutos MD, et al. Utility of Basal Regional Oximetry Saturation for the Diagnosis of Acute Tubular Necrosis in the Early Postoperative Period Following Kidney Transplantation. Transplant Proc. 2019 Mar;51(2):328-333.

7. Grosenick D, Cantow K, Arakelyan K, Wabnitz H, Flemming B, Skalweit A, et al. Detailing renal hemodynamics and oxygenation in rats by a combined near-infrared spectroscopy and invasive probe approach. Biomed Opt Express. 2015 Jan 6;6(2):309-23.

8. Vaughan DL, Wickramasinghe YA, Russell GI, Thorniley MS, Houston RF, Ruban E, et al. Near infrared spectroscopy: Blood and tissue oxygenation in renal ischemia-reperfusion injury in rats. International Journal of Angiology volume 4, 25–30(1995)

Laboratory pig was chosen for our model for several reasons. The pig kidney is very similar to that of a human in anatomy and physiology [9,10]. These similarities extend to vascular supply, capsule structure and organ shape [11,12]. We selected animals weighing approximately 40kg since their organs are similar in size to those of humans. Their internal anatomy and organ placement are also similar to those of humans, allowing us to mimic the physical conditions of human kidney transplantation.

9. Giraud S, Favreau F, Chatauret N, Thuillier R, Maiga S, Hauet T. Contribution of large pig for renal ischemia-reperfusion and transplantation studies: the preclinical model. J Biomed Biotechnol. 2011;2011:532127.

10. Kirk AD. Crossing the bridge: large animal models in translational transplantation research. Immunol Rev. 2003 Dec;196:176-96.

11. Bagetti Filho HJ, Pereira-Sampaio MA, Favorito LA, Sampaio FJ. Pig kidney: anatomical relationships between the renal venous arrangement and the kidney collecting system. J Urol. 2008 Apr;179(4):1627-30.

12. Pereira-Sampaio MA, Favorito LA, Sampaio FJ. Pig kidney: anatomical relationships between the intrarenal arteries and the kidney collecting system. Applied study for urological research and surgical training. J Urol. 2004 Nov;172(5 Pt 1):2077-81.

**OBJECTIVES**

The primary objective of the study was to design, construct and test a NIRS-based renal graft perfusion monitoring system.

The secondary objective was to establish and describe a laboratory animal model of heterotopic autotransplantation, used to mimic conditions (both anatomical and physiological) of human kidney transplantation using up-to-date techniques. This model could be used as a valuable tool for surgical training.

**ETHICAL STATEMENT**

This study was reviewed and approved by the Ministry of Health of the Czech Republic – num. 29/2016.

All legal and ethical requirements for this animal laboratory experiment have been met.

The Institute for Clinical and Experimental Medicine, Prague, Czech Republic (IKEM), is in possession of an active license, which enables it to take part in experimental work on laboratory animals.

All transplantations and procedures performed on laboratory animals were carried out in an operating theatre under the care of a trained anesthesiologist. After surgery, all animals were administered sufficient analgesic drugs. All animals were euthanized under full anesthesia at the end of the experiment.

**STUDY DESIGN**

In-vivo animal testing was divided into two stages and three subgroups of laboratory animals.

The first group (6 animals) was utilized to verify and optimize the technical aspects and functionality of the prototype sensor. The animal was put under full anesthesia, kidney was approached extraperitoneally using midline laparotomy, and the vessel supply of kidney was prepared for clamping. The prototype sensor was placed on the parenchyma of the kidney. The kidney was then put through a series of cycles, during which kidney blood flow (arterial or venous) was obstructed (fully or partially) for 10 minutes and later returned to normal.

The second group (4 animals) was utilized to establish and optimize sensor placement in the closed wound and to verify its function reliability over an extended period of time (48 hours). The animal was put under full anesthesia, kidney was approached extraperitoneally using midline laparotomy. The prototype sensor was placed on the exposed kidney capsule, located on the ventral plane of the organ. The connecting cord was passed through the abdominal wall, the battery and transmitter were fixed to the back of the animal and the wound was closed. The animal was then allowed to move freely for 48 hours.

The third group (20 animals) was utilized to simulate transplantation perioperative and early postoperative conditions and to verify system functionality and efficiency. We prepared a unique laboratory porcine model of kidney transplantation. Under full anesthesia we performed an ipsilateral kidney autotransplantation to the iliac fossa using the retroperitoneal approach – mimicking the conditions of human transplantation. The transplantation was completed with a standard full-thickness ureteroneocystostomy. The prototype sensor strap was then applied to the ventral plane of the graft. The connecting cord was passed through the abdominal wall, and the battery and transmitter were fixed to the back of the animal. We fixed a second sensory system to the contralateral kidney in order to compare the performance of the transplanted graft and native kidney, thus eliminating any system perfusion complications. We performed a 48 hour measurement after transplantation. During this period, the animal was in an awakened state and moving freely. The animal was then put under anaesthesia and underwent short-termed measurements with arterial or venous clamping of the graft, which lasted 10 minutes. All animals were euthanized under full anesthesia at the end of the experiment.

**EXPERIMENTAL PROCEDURES**

ANAESTHESIA PROTOCOL

Sedation: Intramuscular injection of Ketamine+Azaperone+Atropine*.

Venous access: Marginal ear vein – medial or lateral auricular vein.

Anaesthesia:

Intravenous bolus infusion of Propofol+Fentanyl*.

Continuous inhalation of Isoflurane + continuous intravenous infusion of Fentanyl.

Relaxation: Intravenous bolus of Pipecuronium Bromide*.

Antibiotics: Amoxycilline(875mg/125mg) with an enzyme inhibitor.

Arterial access:

Ear arteries – Medial auricular arterial branch or Intermediate auricular arterial branch through percutaneous access by Seldinger technique;

Femoral artery – percutaneous access by Seldinger technique;

Femoral artery – open preparation and approach(Fig 1)

Monitoring: Continuous arterial pressure, ECG, Saturation (tongue, lip)

Euthanasia – under full anaesthesia – Thiopental (1-2g)+ KCl 7,45% solution (40-50ml) i.v.

LOCATION

experimental surgical theatre; laboratory pen

* All drug dosages were calculated based on actual animal weight

**EXPERIMENTAL ANIMALS**

Laboratory pig (breed: Přeštické prase); female; weight 35-41kg (median 37,5kg)

**HOUSING**

Bedless housing with a gradient bed and a grate mud.

Free feeding - feeding twice a day with complete feed mixture in a dose corresponding to the age and weight of the animal

**SAMPLE SIZE**

Group I – 6 animals

Group II – 4 animals

Group III – 20 animals

Since this was our first animal study in development of the NIRS-based monitoring system, we decided to use small animal groups. We aimed for stable and reliable outcomes and system performance. More significant statistical data could be acquired in further studies, if the system proves to be efficient.

**ALLOCATING TO EXPERIMENTAL GROUPS**

No randomisation

**EXPERIMENTAL OUTCOMES**

Primary - We designed and constructed a new NIRS-based measurement system for kidney graft perfusion monitoring. The system proved to be safe for the laboratory animal. The measurement system manifested reliable performance in detecting diminished blood perfusion of transplanted kidneys.

Secondary - we described technical and surgical aspects of a porcine model of heterotopic kidney autotransplantation, using up-to-date techniques, utilized nowadays during human kidney transplantation.

**STATISTICAL METHODS**

**Monitoring system data analysis**

After initial processing, the data were subjected to the classification of detecting diminished blood perfusion. To train the classifier, we used data from both the long-term and short-term measurements. All of the data were used in testing. To avoid overlearning, the leave-one-out cross-validation was used: to evaluate the performance on a chosen measurement, data obtained during this measurement were excluded from a training set. The classifier was retrained using the remaining data, and only then was it applied to the previously excluded measurement, evaluating its success rate.

To classify blood perfusion, we used a Bayesian classifier. The probability density functions (PDF) of the classified data were approximated using a Gaussian mixture model (GMM) using the data from long-term measurements with correctly functioning kidneys. In cases where data were obtained from short-term measurements (i.e. measurements where veins and arteries were mechanically occluded), we approximated PDFs using the kernel density estimation (KDE) with a Gaussian kernel. We selected the kernel density estimation because of its ability to approximate more complex probability density functions. However, in the case of long-term measurements, there is a large amount of data, which makes a KDE based classifier computationally intensive. We therefore chose a GMM in its place.

The classifier was constructed to identify 3 classes:

1. correct blood perfusion
2. diminished blood perfusion due to a vein obstruction
3. decreased blood perfusion due to an artery obstruction

To avoid false-positive detections of diminished blood perfusion due to sensor movement or other short-term interferences, the classifier initiates a warning only if diminished blood perfusion is classified continuously for more than 3 minutes.

**BASELINE DATA+NUMBERS ANALYZED+OUTCOMES AND ESTIMATIONS**

Only animals in group III were included into the data analysis for the laboratory model.

Animal+surgery data

|  | SURGERY TIME (min) | COLD ISCHAEMIA TIME (min) | MANIPULATION TIME (min) | cons. solution perfusion (ml) | animal weight (kg) |
| --- | --- | --- | --- | --- | --- |
| 1 | 195 | 54 | 30 | 250 | 36 |
| 2 | 178 | 42 | 26 | 200 | 38 |
| 3 | 187 | 32 | 32 | 300 | 41 |
| 4 | 160 | 45 | 29 | 250 | 35 |
| 5 | 156 | 30 | 20 | 200 | 37 |
| 6 | 172 | 44 | 25 | 300 | 36 |
| 7 | 168 | 58 | 25 | 300 | 38 |
| 8 | 135 | 50 | 28 | 400 | 40 |
| 9 | 129 | 33 | 23 | 400 | 39 |
| 10 | 150 | 32 | 24 | 150 | 35 |
| 11 | 165 | 55 | 24 | 200 | 35 |
| 12 | 180 | 37 | 20 | 200 | 37 |
| 13 | 150 | 45 | 19 | 200 | 38 |
| 14 | 127 | 24 | 20 | 150 | 36 |
| 15 | 160 | 37 | 21 | 300 | 39 |
| 16 | 131 | 37 | 25 | 200 | 41 |
| 17 | 125 | 42 | 22 | 250 | 38 |
| 18 | 142 | 26 | 19 | 200 | 36 |
| 19 | 135 | 31 | 22 | 300 | 37 |
| 20 | 123 | 27 | 20 | 300 | 39 |
| median | 153 | 37 | 23,5 | 250 | 37,5 |
| SD | 22,14 | 10,01 | 3,82 | 71,58 | 1,87 |

We obtained 14 long-term measurement data sets. In other cases, data were lost due to a technical failure or an acute thrombosis of the graft, which occurred during monitoring.

**Data analysis - 14 long-term (48hours) renal graft perfusion measurements**

| Vein obstruction FPR | 0.20% |
| --- | --- |
| Artery obstruction FPR | 0.30% |
| Combined FPR | 0.30% |

We obtained 18 artery obstruction and 16 vein obstruction short-term data sets.

**Data analysis – short-term measurements – iatrogenic clamping of renal graft arteries or veins**

|  | n | Detection FNR | Classification FNR | sensitivity |
| --- | --- | --- | --- | --- |
| Artery obstruction | 18 | 0.0% | 5,6% | 100.0% |
| Vein obstruction | 16 | 0.0% | 31,3% | 100.0% |

**ADVERSE EVENTS**

Three grafts had an early graft failure due to an early arterial thrombosis; in the first two cases, the grafts had their artery anastomosed onto a thin external iliac artery. We modified the technique of the transplantation afterwards – anastomosing our graft arteries to the suprailiac region of the abdominal aorta. The third thrombosis occurred on a graft with complex arterial anatomy with implanted pole artery.

**INTERPRETATION**

We designed and constructed a new NIRS-based measurement system for kidney graft perfusion monitoring. Under laboratory conditions, our NIRS monitoring system proved reliable, with a very high degree of sensitivity as well as favorable specificity between arterial and venous causes. The system can be a useful tool for providing an early diagnosis of diminished blood perfusion of kidney transplants during the early postoperative period. However, further testing is still needed.

We described technical and surgical aspects of a porcine model of heterotopic kidney autotransplantation, using up-to-date techniques, utilized nowadays during human kidney transplantation. Our experimental model demonstrated that a pig laboratory model is a useful and valuable tool for surgical training. It can help to shorten the operation times and lower the complication rates. The model can also be extended to serve not only as a simple training tool for surgical techniques. Considering pig to human similarities in physiology, biochemistry, and immunology, it can also be used as a short or long term model for kidney transplantation.

As for the limitations of the study, the low number of animals may be the biggest issue for our monitoring system data analysis. If aiming for significant statistical data, the number of laboratory animals would have to be higher. Regarding the animal transplantation model, the price is the main limitation when animal models are used for purely training purposes.

**TRANSLATION**

We believe the application of our monitoring system in current human transplantation medicine is feasible, and our prototype is prepared for human testing.

A similar system might also be of great use in various fields of surgery (e.g., transplantation of other parenchymatous organs, muscle flap transfers, etc.).

Animal models prove to be useful training tools for young surgeons. Our specific model could lead to a shortened surgery and manipulation time and result in a better kidney graft performance after transplantation.

FUNDING

Ministry of Health of Czech Republic - **AZV ČR**; Czech Health Research council; Grant 16-34134A, 2016
